# Cost of stay and characteristics of patients with stroke and delayed discharge for non-clinical reasons

**DOI:** 10.1038/s41598-022-14502-5

**Published:** 2022-06-27

**Authors:** Amada Pellico-López, Ana Fernández-Feito, David Cantarero, Manuel Herrero-Montes, Joaquín Cayón-de las Cuevas, Paula Parás-Bravo, María Paz-Zulueta

**Affiliations:** 1 Cantabria Health Service, Avda. Derechos de la Infancia, 31. C.P., 39340 Suances, Cantabria Spain; 2 Departamento de Enfermería, Universidad de Cantabria, Avda. Valdecilla s/n. C.P., 39008 Santander, Cantabria Spain; 3 Departamento de Medicina, Facultad de Medicina y Ciencias de la Salud, Universidad de Oviedo, Avda. Julián Clavería s/n C.P., 33006 Oviedo, Principado de Asturias Spain; 4 Área de Investigación en Cuidados, Grupo de Procesos Asistenciales de Enfermería, Instituto de Investigación Sanitaria del Principado de Asturias (ISPA), Avda del Hospital Universitario, s/n. C.P., 33011 Oviedo, Principado de Asturias Spain; 5 Departamento de Economía, Universidad de Cantabria, Avda. de los Castros s/n C.P., 39005 Santander, Cantabria Spain; 6 IDIVAL, Research Group of Health Economics and Health Services Management–Research Institute Marqués de Valdecilla C/ Cardenal Herrera Oria s/n., 39011 Santander, Cantabria Spain; 7 IDIVAL, Grupo de Investigación en Enfermería, C/ Cardenal Herrera Oria s/n. C.P., 39011 Santander, Cantabria Spain; 8 Departamento de Derecho Privado, Universidad de Cantabria, Avda. de los Castros s/n. C.P.,, 39005 Santander, Cantabria Spain; 9 IDIVAL, Grupo de Investigación en Derecho Sanitario y Bioética, GRIDES, C/ Cardenal Herrera Oria s/n. C.P., 39011 Santander, Cantabria, Spain

**Keywords:** Geriatrics, Health care economics, Health services

## Abstract

Delayed discharge for non-clinical reasons (bed-blocking) is characteristic of pathologies associated with ageing, loss of functional capacity and dependence such as stroke. The aims of this study were to describe the costs and characteristics of cases of patients with stroke and delayed discharge for non-clinical reasons (bed-blocking) compared with cases of bed-blocking (BB) for other reasons and to assess the relationship between the length of total stay (LOS) with patient characteristics and the context of care. A descriptive cross-sectional study was conducted at a high complexity public hospital in Northern Spain (2007–2015). 443 stroke patients presented with BB. Delayed discharge increased LOS by approximately one week. The median age was 79.7 years, significantly higher than in cases of BB for other reasons. Patients with stroke and BB are usually older patients, however, when younger patients are affected, their length of stay is longer in relation to the sudden onset of the problem and the lack of adequate functional recovery resources or residential facilities for intermediate care.

## Introduction

At the beginning of the twenty-first century, neurological diseases such as stroke remain the most important cause of disability-adjusted life years lost worldwide and the second leading cause of death after cardiovascular diseases^1^. However, a decrease in stroke mortality is observed, especially in younger population groups and in higher-income countries, mainly due to the control of cardiovascular risk factors such as hypertension and smoking^1,2^. In northern Spain, one of the most ageing regions in Europe, stroke also remains the leading cause of death in women, and the second in the population as a whole^3^. This is related to the progressive ageing of the population and the fact that women live the longest. Life expectancy was 85.6 years in women compared to 79.74 years in men in Northern Spain in 2015 ^4^. In Spain, the same trend of decreasing stroke mortality has also been observed^3^. Despite higher mortality in women, the impact as the main cause of disability has been found to affect men to a greater degree^5^.

Early rehabilitation after stroke reduces mortality, improves functional prognosis, favors the patient's return to his or her previous environment, and reduces the overall costs of the disease. As part of the care strategy, it is recommended that coordination between levels of care be improved to facilitate the patient's recovery by facilitating access to comprehensive rehabilitation as soon as possible^6^.

Pathologies such as stroke, associated with population aging, loss of functional capacity and increased dependency on another person for self-care, are associated with the phenomenon of delayed discharge for non-clinical reasons, known in the literature as bed-blocking (BB)^7^. This phenomenon refers to the situation in which a patient remains admitted for non-medical reasons despite being considered ready for medical discharge from the hospital^8^, representing a failure in discharge planning and a lack of availability of intermediate care resources that are alternative to acute hospitalization^9^. Furthermore, BB is related to the need for functional recovery and rehabilitation following acute hospitalization^10,11^.

Regarding the number of users affected by BB, significant variations have been found worldwide depending on the study context, with a prevalence ranging from 1.6% to 91.3% and with a significant cost despite the fact that its economic analysis has been scarcely addressed^12^. The United Kingdom is the country that has conducted the most studies to research this problem, which is continuously monitored and is considered a key indicator by the National Health Service. Recent records published in Scotland report that the number of stays affected by delayed discharge were 8.5% in 2018/2019, with an increasing trend compared to the previous period^13^. The BB phenomenon appears in any healthcare system, both in those with universal coverage funded by taxes and those funded by private insurance, such as in the United States^12^. It has negative consequences beyond the inefficient and inappropriate use of the acute hospital bed resource, such as increased risk of infections related to hospital stay, adverse events such as falls or medication errors, death, loss of functional capacity and negative emotional impact on patients and families who suffer from this situation^8,14^.

Therefore, the study of this phenomenon is relevant in patients with stroke and who are at an increased risk of complications due to their advanced age and acquired disability. It is worthwhile to determine the differential characteristics of cases with stroke compared with other patients with BB because these are patients who live in the community and are hospitalized for an acute problem that causes dependence and the need for subsequent rehabilitation. Given that stroke is a highly relevant problem in terms of BB^7^, it is necessary to know what is distinctive about these cases compared to other BB cases, in terms of characteristics such as sex, age, length of hospital stay and the relationship of stay with external variables, such as destination at discharge.

The aims of the present study were to describe the costs and characteristics of cases with stroke and BB compared with cases of BB for other reasons and to assess the relationship between LOS and patient characteristics and the context of care.

## Material and methods

### Design

A descriptive, observational, cross-sectional study was conducted based on the analysis of the Minimum Basic Hospital Discharge Data Set (MBDS) of delayed discharge cases registered between January 1, 2007 and December 31, 2015 by the hospital admission service.

### Setting and sample

The study setting was the Valdecilla Hospital in Cantabria (Northern Spain), a high-complexity public hospital. This center had 903 inpatient beds in 2015^15^ and directly served a population of 319,751 users. The Valdecilla Hospital is a hospital of reference for two other local hospitals with a catchment area population of 255,000 people. This center is accredited as a teaching center and is a national reference for certain highly qualified healthcare and technological services^16^.

The study population was the total number of cases with BB during a nine-year study period of all patients discharged from the 25 medical and surgical services of the hospital. The study included all those patients identified as ready for medical discharge by the hospital's admission department, but whose actual discharge was delayed by more than 24 h. A patient is considered ready for medical discharge when hospital's admission department receives the hospital discharge report^17^ from the doctor in charge of each care process of a patient. Of the total number of BB cases found, two groups were formed, patients affected by stroke and the remaining cases. Patients discharged to other hospitals or in charge of the hospital's own home hospitalization service were excluded.

### Measures

The data for the study were collected thanks to the information provided by the hospital's Admission and Analytical Accounting (AA) Services. The information was based on the MBDS of the cases. The Diagnostic Related Groups (DRGs) included in the stroke group were, according to DRG coding version 25.0, those that were in force at the end of the study period^18^: DRG 14 (stroke with infarction), DRG 15 (non-specific stroke and precerebral occlusion without cerebral infarction), DRG 532 (transient ischemic attack, precerebral occlusions, seizures and headache with complications), DRG 533 (other nervous system disorders except transient ischemic attack, seizures and headache with complications), DRG 810 (intracranial hemorrhage), DRG 832 (transient cerebral ischemia) and DRG 880 (acute ischemic attack with use of thrombolytic agent).

Among the variables collected, we differentiated between those related to length of stay and its associated costs, those of the patient and those of the context of care. Regarding the length of stay, we calculated length of appropriate stay (LAS) or days between the date of admission and medical discharge, length of prolonged stay (LPS) or days between the date of medical discharge and actual discharge, and length of total stay (LOS), the sum of the above. The cost of length of appropriate stay is the product of the days of appropriate stay multiplied by the cost of stay per DRG. The cost of length of prolonged stay is the product of the days of prolonged stay cost multiplied by the cost of stay in the hospitalization unit. The cost of length of total stay is the sum of the above.

In relation to the patient, the variables assessed were age, sex, and relative weight of the DRG to determine the complexity of the process. The weight of the DRG translates the complexity in terms of consumption of hospital resources to attend its patients, based on the average annual cost of hospitalization in acute units (weight = 1)^19^. Regarding the context of care, we recorded the type of admission (urgent or programmed), urban or rural place of residence (urban corresponding to residents in the same region as the hospital and with more than 50,000 inhabitants and with a density of more than 1,500 residents per km2, rural to the rest of the regions), year of discharge and discharge destination (long-stay center arranged for functional recovery, home, death during the period of prolonged stay and residential center for dependent persons). The difference between the total length of stay of the cases of delayed discharge found and those which would have corresponded for the same DRG and year of discharge according to hospital data was calculated based on the hospital's own data.

### Statistical analysis

All data were analyzed using R 4.1.2 for Windows. In the descriptive analysis, proportions with their corresponding 95% confidence intervals (95% CI) were estimated for discrete variables. For continuous variables, the median and interquartile range are reported. To compare the differential characteristics of the stroke patient groups with the rest of the BB cases, continuous quantitative variables were compared using the Mann Whitney test and categorical variables were compared using Pearson's chi-squared test (χ^2^). An adjustment was made for multiple comparisons applying the Bonferroni correction, considering a p value less than or equal to 0.0015 as significant. Using total length of stay in days as the dependent variable, its relationship with the independent variables of patient characteristics and context of care was assessed using linear regression models. To ensure normality of the residuals, the total length of stay was transformed using a logarithm. Estimates, 95% CI and p values are reported. A p value of less than 0.05 is considered significant.

### Ethics approval

The research protocol was approved by the Clinical Research Ethics Committee of Cantabria (internal code 2015.085) and was conducted in accordance with the approved guidelines. The need for informed consent was waived by ethics committee of Cantabria as it was a retrospective study.

## Results

The descriptive data of the cohort are published elsewhere^20^. A total of 443 patients with a diagnosis of stroke and BB were identified during the study period. These patients accounted for 0.53% (95%CI 0.48–0.58) of the total number of patients discharged during the same period with the same DRGs.

Of the total 12,084 days of LOS, 3,512 days corresponded to LPS. A total of 24.1% (95%CI 20.24- 28.42) of cases had a prolonged length of stay of only one day. The characteristics of these cases in terms of LOS and associated costs are shown in Table 1. The median LOS duration was 22 days, with a median of length of stay of four days after the day of medical discharge (LPS). The median length of stay that would have corresponded to the same DRG and year of discharge for each case had BB not occurred was 9.13 days (Q1 1.78; Q3 20.95), which is more than double the median LOS duration of the cases.

**Table 1:** Comparison of cases with stroke versus other cases of delayed discharge for non-clinical reasons: length of stay and costs. Cantabria (Northern Spain), 2007–2015.

	Stroke (n = 443)	No Stroke (n = 2,572)	p-value
	Median [Q1; Q3]	Median [Q1; Q3]	
LOS^a^ (days)	22.0 [16.0; 33.5]	21.0 [12.0; 35.0]	*0.004*
LAS^b^ (days)	17.0 [11.0; 23.0]	15.0 [8.0; 27.0]	*0.032*
LPS^c^ (days)	4.0 [2.0; 9.0]	3.0 [1.0; 7.0]	< *0.001*
Cost of LOS^a^ (€)	12,100 [7,920; 18,300]	10,100 [5,210; 18,500]	< *0.001*
Cost LAS^b^ (€)	9,830 [6,390; 15,400]	7,930 [3,840; 16,400]	< *0.001*
Cost of LPS^c^ (€)	1,230 [550; 2,730]	933 [311; 2,160]	*0.001*

^a^LOS, Length of total stay;

^b^LAS, Length of appropriate stay;

^c^LPS, Length of prolonged stay.

The cost of these cases amounted to a total of €6,433,208.26, of which €1,017,763.26 corresponded to the LPS. The median LOS cost of the cases was €12,100. Divided into two periods, the median cost corresponding to LAS was €9,830 and that of LPS was €1,230. Both length of stay and the corresponding cost were significantly higher in stroke cases.

Of the stroke cases with delayed discharge, 53.3% were women. The mean age of this patients was 80.22 years, ranging between 45 and 103 years. Median age was 79.7 (Q1 70.96; Q3 85.78). Up to 22.6% (95%CI 18.76- 26.76) were younger than 75 years. Comparing stroke cases with BB versus all other BB cases, the former were older (p < 0.001). In the group of patients with stroke and BB, females were significantly older (p < 0.001) with a median age of 83.7 years (Q1 79.15; Q3 87.72) versus 79.4 years (Q1 70.93; Q3 85.23) in males. The median DRG weight was 2.56 (Q1 1.87; Q3 4.43). The median DRG weight was significantly higher in the group of BB patients with stroke, compared to a median of 2.21 (Q1 1.38; Q3 3.61), and with a range between 0.81 and 4.79.

The characteristics of these cases and the context of care are shown in Table 2. Regarding the characteristics of the context of care, in cases of stroke with BB, 77.6% resided in the urban area in which the hospital is located. Comparing stroke cases with BB versus the rest of the BB cases, a higher proportion of stroke cases were admitted urgently (p < 0.001), with 97.9% of cases admitted unexpectedly. A total of 79.0% of stroke cases with BB were discharged to a long-stay facility for functional recovery. 2008 was the year with the highest number of cases.

**Table 2:** Comparison of cases with stroke versus other cases of delayed discharge for non-clinical reasons: characteristics of the patient and the context of care. Cantabria (Northern Spain), 2007–2015.

		Stroke (n = 443)		No Stroke (n = 2,572)		*p-value*
		n (%)	95%CI^a^	n (%)	95%CI^a^	
Sex	Male	207 (46.7%)	(42.00–51.94)	1237 (48.09%)	(46.15- 50.05)	*0.631*
	Female	236 (53.3%)	(48.51- 57.99)	1335 (51.90%)	(49.95- 53.85)	
Place of residence	Rural	99 (22.3%)	(18.55- 26.52)	579 (22.51%)	(20.91- 24.18)	*0,988*
	Urban	344 (77.6%)	(73.48- 81.45)	1993 (77.49%)	(75.82- 79.09)	
Type of hospitalization	Programmed	9 (2.03%)	(0.93- 3.82)	202 (7.85%)	(6.84- 8.96)	< *0,001*
	Urgent	434 (97.97%)	(94.18- 99.07)	2370 (92.15%)	(91.04- 93.16)	
Destination at discharge	Long-Term Care Center	350 (79.01%)	(74.91- 82.71)	2027 (78.81%)	(77.18- 80.38)	*0,180*
	Home	68 (15.35%)	(12.12- 19.05)	344 (13.37%)	(12.08- 14.75)	
	Deceased	24 (5.42%)	(3.50- 7.95)	174 (6.76%)	(5.82- 7.80)	
	Other^b^	1 (0.27%)	(0.01- 1.25)	27 (1.05%)	(0.69- 1.52)	
Year of medical discharge	2007	62 (14.0%)	(10.90- 17.58)	312 (12.1%)	(10.89- 13.45)	*0,036*
	2008	75 (16.9%)	(13.56- 20.75)	372 (14.5%)	(13.13- 15.88)	
	2009	53 (11.9%)	(9.09- 15.36)	320 (12.4%)	(11.19- 13.78)	
	2010	65 (14.7%)	(11.51- 18.32)	301 (11.7%)	(11.19- 13.78)	
	2011	60 (13.5%)	(10.49- 17.09)	336 (13.1%)	(11.78- 14.43)	
	2012	45 (10.2%)	(7.51- 13.36)	247 (9.6%)	(8.49- 10.81)	
	2013	35 (7.9%)	(5.56- 10.82)	243 (9.4%)	(8.34- 10.64)	
	2014	25 (5.6%)	(3.68- 8.22)	199 (7.4%)	(6.73- 8.84)	
	2015	23 (5.2%)	(3.32- 7.69)	242 (9.4%)	(8.31- 10.60)	

^a^95%CI, 95% confidence interval;

^b^nursing homes for people with dependency.

Univariate analyses are shown in Table 3. Using the duration in days of LOS as the dependent variable, in the group of stroke patients who experienced BB, a statistically significant association (*p* < 0.05) was found with the independent variables: age (longer stay in patients younger, *p* < 0.001), DRG weight (longer with high complexity p < 0.001), type of hospitalization (longer in emergency admission, *p* = 0.011), discharge destination (longer to home, *p* < 0.001) and year of discharge (increasing LOS in 2008 and decreasing LOS in 2011). In the group of other patients with BB, a statistically significant association (*p* < 0.05) was found with the independent variables: age (longer stay in patients younger, p < 0.001), DRG weight (longer with high complexity *p* < 0.001), discharge destination (longer to home and others as nursing care home, *p* < 0.001) and year of discharge (increasing LOS in 2008 and decreasing LOS from 2010 to 2015).

**Table 3:** Relationship between LOS^a^ by patient characteristics and context of care in patients with stroke and delayed discharge for non-clinical reasons: univariate analyses. Cantabria (Northern Spain), 2007–2015.

		Stroke (n = 443)			No Stroke (n = 2,572)		
		Total stay (days)		*p-value*	Total stay (days)		*p-value*
		Estimate	^b^95%CI		Estimate	^b^95%CI	
Gender	Male	Reference			Reference		
	Female	− 0.08	(− 0.19; 0.03)	*0.161*	− 0.05	(-0.11; 0.02)	*0.140*
Age (years)		− 0.02	(-0.02; − 0.01)	< *0,001*	− 0.01	(-0.01; 0.00)	< *0,001*
DRG^c^ Weight^d^		0.10	(0.06; 0.15)	< *0,001*	0.04	(0.03; 0.04)	< *0,001*
Type of hospitalization	Planned	Reference			Reference		
	Emergency	0.17	(0.04; 0.31)	*0.011*	0.06	(− 0.02; 0.14)	*0.122*
Place of residence	Rural	Reference			Reference		
	Urban	− 0.22	(− 0.62; 0.17)	*0.265*	*− 0.06*	*(-0.18; 0.06)*	*0.324*
Discharge destination	Long-term care	Reference			Reference		
	Home	0.31	(0.16; 0.47)	< *0,001*	0.42	(0.32; 0.51)	< *0,001*
	Death	0.04	(− 0.21; 0.28)	*0.764*	− 0.02	(− 0.15; 0.11)	*0.763*
	Others^e^	0.29	(− 0.87; 1,45)	*0.624*	0.60	(0.29; 0.91)	< *0,001*
Year of discharge	2007	Reference			Reference		
	2008	0.20	(0.01; 0.40)	*0.043*	0.23	(0.11; 0.35)	< *0,001*
	2009	− 0.10	(− 0.32; 0.11)	*0.347*	− 0.08	(− 0.21; 0.05)	*0.261*
	2010	− 0.20	(− 0.40; 0.01)	*0.059*	− 0.26	(− 0.38; − 0.13)	< *0,001*
	2011	− 0.32	(− 0.53; − 0.12)	*0.002*	− 0.20	(− 0.32; − 0.07)	*0.002*
	2012	− 0.05	(− 0.27; 0.17)	*0.644*	− 0.24	(− 0.37; − 0.10)	*0.001*
	2013	− 0.19	(− 0.43; 0.05)	*0.127*	− 0.17	(− 0.30; − 0,03)	*0.019*
	2014	− 0.04	(− 0.31; 0.23)	*0.794*	− 0.22	(− 0.37; − 0.08)	*0.003*
	2015	− 0.17	(− 0.45; 0.10)	*0.220*	− 0.13	(− 0.26; 0.01)	*0.070*

^a^LOS: length of total stay;

^b^95%CI, 95% confidence interval;

^c^DRG, diagnosis-related group;

^d^DRG weight, complexity in terms of consumption of hospital resources for care provision, based on the average annual cost of hospitalization in acute care units (weight = 1);

^e^nursing homes for people with dependency.

Multivariate analyses are shown in Table 4. There was no multicollinearity problem. The patient factors finally determining LOS in stroke cases with BB (p < 0.001), were younger age, higher DRG weight and discharge destination to home (which lengthens the LOS). Moreover, LOS was reduced in the years 2010 and 2011 and in the cases that were discharged to nursing homes for dependent people. In the remaining cases, the greater weight of the DRG and both discharge destination to home and other destinations such as nursing care homes were determinants of longer LOS. Both the increase in LOS in 2008 and the reduction from 2010 to 2014 remained significant.

**Table 4:** Relationship between LOS^a^ by patient characteristics and context of care in patients with stroke and delayed discharge for non-clinical reasons: multivariate analyses. Cantabria (Northern Spain), 2007–2015.

		Stroke (n = 443)			No Stroke (n = 2,572)		
		LOS (days)		*p-value*	LOS (days)		*p-value*
		Estimate	^b^95%CI		Estimate	^b^95%CI	
Age (years)		-0.01	(-0.02; -0.01)	< *0,001*	− 0.00	(-0.00; 0.00)	*0.184*
DRG^c^ Weight^d^		0.09	(0.05; 0.13)	< *0,001*	0.04	(0.03; 0.04)	< *0,001*
Discharge destination	Long-term care	Reference			Reference		
	Home	0.22	(0.07; 0.37)	*0,005*	0.33	(0.24; 0.42)	< *0,001*
	Death	− 0.03	(-0.26; 0.19)	*0.766*	− 0.02	(-0.14; 0.10)	*0.776*
	Others^e^	− 0.03	(-1.12; 1,06)	*0.961*	0.46	(0.16; 0.76)	*0,003*
Year of discharge	2007	Reference			Reference		
	2008	0.16	(-0.03; 0.34)	*0.097*	0.20	(0.08; 0.31)	*0,001*
	2009	− 0.15	(-0.35; 0.05)	*0.154*	− 0.07	(-0.19; 0.05)	*0.244*
	2010	− 0.20	(-0.39; − 0.01)	*0.040*	− 0.23	(-0.35; − 0.11)	< *0,001*
	2011	− 0.31	(-0.51; − 0.12)	*0.002*	− 0.19	(− 0.30; − 0.07)	*0.002*
	2012	− 0.08	(-0.29; 0.13)	*0.434*	− 0.20	(− 0.33; − 0.08)	*0.002*
	2013	− 0.17	(-0.39; 0.06)	*0.150*	− 0.17	(− 0.30; − 0,04)	*0.010*
	2014	− 0.07	(-0.32; 0.19)	*0.607*	− 0.18	(− 0.31; − 0.04)	*0.011*
	2015	− 0.14	(-0.40; 0.12)	*0.299*	-0.12	(− 0.25; 0.01)	*0.065*

^a^LOS: length of total stay;

^b^95%CI, 95% confidence interval;

^c^DRG, diagnosis-related group;

^d^DRG weight, complexity in terms of consumption of hospital resources for care provision, based on the average annual cost of hospitalization in acute care units (weight = 1);

^e^nursing homes for people with dependency.

## Discussion

After classifying the 3,015 total BB cases found in the study period^20^ by pathology, the cases of patients admitted with a diagnosis of stroke represented the largest group. The literature identifies stroke and nervous system diseases in general as a pathology of special risk for delayed discharge. This fact is related to the supervening disability that makes it difficult for the patient to return to the usual environment after hospitalization^7,10,11,21,22^. However, compared with the total number of stroke cases discharged during the study period, cases of stroke with BB accounted for a very small proportion of all cases. The literature on this subject shows considerable variations in prevalence depending on the context^12^, in our case being the total number of discharges from all hospital departments over a nine-year period. Furthermore, in our context, the data on the duration of hospital stays suggest a possible problem of underdiagnosis.

In our study, the days of prolonged stay in stroke cases due to delayed discharge for non-clinical reasons accounted for a quarter of the total length of stay. However, considering the length of stay that would have corresponded to a similar case with the same DRG and year of discharge, the LOS is more than double. Our results suggest a covert delayed discharge, since medical discharge does not depend on objective criteria based solely on clinical criteria, and therefore both the number of cases of delayed discharge for non-clinical reasons and their prolonged length of stay would most likely be greater. The UK National Health Service sets clear criteria for when a patient is considered ready to go home from an inpatient resource: a decision by the clinician that the patient is ready to go home (in acute inpatient settings), or a decision by the multidisciplinary care team (in the case of chronic inpatient settings) and whether such discharge is considered safe for the patient. These criteria depend on whether the patient requires inpatient care, but not whether the patient has regained his or her previous level of function^23^. For an accurate measure of length of stay in cases of delayed discharge, a true record of the date of discharge is important, specifying the clinical criteria^24^.

In our study, 15.8% of the total cost of the stay of stroke cases depends on the cost of prolonged stay. The length of appropriate, prolonged and total stay and also the costs were significantly higher in the case of stroke cases compared to the total number of cases of delayed discharge for non-clinical reasons, as is the case with the complexity according to DRG weight. The median DRG weight found was 2.56, which translates into a relatively high complexity based on a reference patient weight = 1^19^. These variables are related, since the greater the weight of the DRG, the higher the stay and cost, as a greater number and greater complexity of diagnostic and therapeutic procedures are required. The cost of hospital treatment in stroke cases depends essentially on the stay at the hospital ward and the diagnostic procedures performed, consisting of laboratory tests and diagnostic imaging. Surgical procedures or intensive care are much less frequent and therefore have less influence on the cost of the process^25^. In general, this complexity reflected in the weight of the DRG is related to longer lengths of stay^26^, however, in our cases, we found a wide range between 0.81 and 4.79. Cases with complexity lower than 1, which would be apparently simple cases but with delayed discharge, could have been admitted for social problems, with similar results found in cases of hospitalization of homeless people^27^.

In our study, we did not find differences in the proportion of men and women between patients with delayed discharge with and without stroke, nor was gender related to longer length of stay. A significant difference in age according to gender appears in those stroke patients affected by delayed discharge for non-clinical reasons, with women being older than men. This could be due to the pathology itself, since national studies show a higher incidence of stroke in men, which decreases with age in those over 85 years^3^. Moreover, although the role of gender in delayed discharge for non-clinical reasons is inconclusive^7,26,28^, the occurrence of differential delayed discharge in two profiles of young men or older women may reflect different causes in each gender related to the lack of caregiver or social support, which are factors related to the problem^10,29–31^. To explain the profile of women, when consulting other studies, we found a relationship between longer hospital stays when patients who previously lived alone were admitted, and it was more likely that these patients were women, of advanced age, with a good previous functional and cognitive level, and without a spouse or caregiver^32^. In addition, women may be more likely to be referred to a residential care facility for dependent persons^7^. Furthermore, to explain the profile of the men, we know that, in our environment, family caregivers are traditionally women, generally the wife or daughter, with a low level of education and working as housewives^33^. The profile of cases in younger men could be explained by a lack of caregiver or family support when the situation of loss of functional capacity related to the reason for admission is unexpected.

In our study, the mean age was 80.22 years, which was significantly higher in patients with stroke than in other patients. Numerous studies in the literature coincide in highlighting the greater probability of suffering BB at an older age^7,21,34,35^ specifically, the relationship between the lengthening of hospital stay in patients with non-traumatic brain injury and older age^36^. However, we found a wide age range, 45 and 103 years, and a longer total length of stay in younger patients affected by delayed discharge. We found other studies that relate younger age in delayed discharge with longer length of stay^26,37,38^. Younger patients may have greater difficulty being admitted to long-stay convalescent or rehabilitation facilities after discharge, and have more complex care needs, as pathologies involving loss of functional capacity are less likely to occur in young people and, because of their unexpectedness, these may be more difficult to assume by the family care network.

Regarding the context of care, most of our cases resided in the urban area coinciding with the district where the hospital is located. Other authors found longer stays in residents in the same area as the hospital^26^, this could be explained by the use of the hospital itself as a temporary stay resource due to the lack of social and health services. However, in the same urban area as the study, there is a long-stay center for functional recovery, which was the most common discharge destination in cases of stroke with BB. The destination at discharge was related to the length of stay in our cases, although the literature consulted provides evidence that in the case of stroke, the availability of long-stay recovery centers in the patients' area of residence does not determine their post-discharge referral, depending more on factors such as age or the complexity of the patient's condition^7^. Planning communication at discharge with the resource that will subsequently receive the patient, regardless of whether it is a residential center or the community, has proven to be an effective measure to promote continuity of care^39^, avoid BB and reduce LOS^14^. Such communication should be standardized, possibly via the help of a liaison professional or by using technological means^14,39^. In our cases, stroke patients who returned home after BB had significantly higher LOS. To favor the preparation of patients and their families for discharge home, we recommend assessing factors such as self-care skills, functional level, caregiver support, presence of symptoms, adherence to prescriptions, the need to adapt the home to make it safer, or relationship with community resources to favor discharge^40^.

Practically all the cases found of patients with stroke and BB were admitted urgently, which is to be expected, as a pathology of unpredictable onset. There were few cases of scheduled admissions. On reviewing the characteristics of these cases, these are relatively young cases, with a mean age of 70.07 years, of heterogeneous etiology, both hemorrhagic and ischemic, and were patients who were admitted by the neurology service who did not require surgery. The hospital under study is a national reference hospital for certain services of high healthcare and technological qualification, with these cases being classified as programmed admissions, referrals from other hospitals in the same region of lesser complexity that cases requiring more complex studies or therapeutic procedures. In the multivariate analysis, there was a discrete significance in favor of a longer stay in cases admitted urgently, probably due to the unexpectedness of the event for the caregivers involved in the patient's care.

The highest number of cases were recorded in 2008, with a certain downward trend only in 2010 and 2011, different from what occurs in the rest of the BB cases with higher evidence that the total length of stay decreases from 2010 to 2015. This result of the remaining BB cases is consistent with the progression found by the authors in terms of the total sample^20^ and with studies that demonstrate the effect of the implementation of the System of Care for Dependent Persons in Spain on hospital stay^41^. However, it appears that stroke cases were not as sensitive to such organizational changes.

As strengths, studies on delayed discharge for nonclinical reasons in Spain are scarce and those found only focus on clinical^21^ or socioeconomic^41^ variables, without relating both fields as in the present study. The study spans a total of nine years, coinciding with the introduction of the dependency care system and the economic recession, and in all the units of a highly complex hospital in one of the most aged regions of the country^42^.

Regarding the limitations of this study, the variables analyzed are based on data collected through the MBDS. This system collects variables at hospital discharge in a systematic, homogeneous, and objective manner. Demographic, clinical (DRG), type of care or social context data were collected, which could be related to delayed discharge for strictly non-clinical reasons. The use of these MBDS records, in addition to guaranteeing systematically collected data, enabled us to handle a large amount of data from a wide period. However, in the process of patient care, other variables that have been shown to be related to the problem, such as lack of social or family support, are collected in the patient's clinical history^10,21,29^, previously residing alone^39^ or increasing their level of dependence for self-care^7,10,11,35^. These data are not objectively reflected in the MBDS, rather, this kind of data is lost and requires a dedicated review of the information recorded by the professionals in the patient’s clinical history. Future lines of research may analyze this phenomenon more in depth by comparing stroke cases who have suffered delayed discharge with controls without delayed discharge in terms of demographic and clinical variables, to identify possible risk factors. Analytical Accounting through Diagnostic Related Group (DRG) does not allow to differentiate the cost reduction as the appropriate stay progresses. This would be possible thanks to the current cost-per-patient accounting systems, which were not available in our setting in the period to which the study refers.

## Conclusions

The prolonged length of stay in stroke cases with delayed discharge for non-clinical reasons increases the cost of stay and accounts for a quarter of the length of stay and can be as much as double the length of stay that would have corresponded for the same DRG and year of discharge had there been no bed-blocking. Patients with stroke have longer length of stay, undergo greater complexity of care and incur a higher cost than other BB cases.

Patients with stroke and BB are usually older patients, however, when younger patients are affected, their length of stay is longer in relation to the sudden onset of the problem and the lack of adequate functional recovery resources or residential facilities for intermediate care.

Patients with stroke and BB are usually admitted on an emergency basis and if they are discharged to their usual home rather than to a long-stay facility for functional recovery, definitive discharge is further delayed.

## Data Availability

The datasets generated during and/or analysed during the current study are available from the corresponding author on reasonable request.
